# Design and Comprehensive Characterization of Tetramethylpyrazine (TMP) for Targeted Lung Delivery as Inhalation Aerosols in Pulmonary Hypertension (PH): In Vitro Human Lung Cell Culture and In Vivo Efficacy

**DOI:** 10.3390/antiox10030427

**Published:** 2021-03-11

**Authors:** Priya Muralidharan, Maria F. Acosta, Alexan I. Gomez, Carissa Grijalva, Haiyang Tang, Jason X.-J. Yuan, Heidi M. Mansour

**Affiliations:** 1 College of Pharmacy, The University of Arizona, Tucson, AZ 85721, USA; priyam@pharmacy.arizona.edu (P.M.); acosta@pharmacy.arizona.edu (M.F.A.); alexan.gomez@medtronic.com (A.I.G.); cari10g@email.arizona.edu (C.G.); 2 Department of Biomedical Engineering, The Arizona State University, Phoenix, AZ 85287, USA; 3 Department of Medicine, Division of Translational & Regenerative Medicine, College of Medicine, The University of Arizona, Tucson, AZ 85721, USA; haiyangtang@email.arizona.edu (H.T.); jxyuan@health.ucsd.edu (J.X.-J.Y.); 4 Department of Medicine, University of California, San Diego, La Jolla, CA 92093, USA; 5 The BIO5 Research Institute, The University of Arizona, Tucson, AZ 85721, USA; 6 Institute of the Environment, The University of Arizona, Tucson, AZ 85721, USA

**Keywords:** respiratory drug delivery, ligustrazine, Rho/ROCK inhibitor, antioxidant, Nrf2, lung vascular disease, advanced spray drying, monocrotaline (MCT), rat model

## Abstract

This is the first study reporting on the design and development innovative inhaled formulations of the novel natural product antioxidant therapeutic, tetramethylpyrazine (TMP), also known as ligustrazine. TMP is obtained from Chinese herbs belonging to the class of *Ligusticum*. It is known to have antioxidant properties. It can act as a Nrf2/ARE activator and a Rho/ROCK inhibitor. The present study reports for the first time on the comprehensive characterization of raw TMP (non-spray dried) and spray dried TMP in a systematic manner using thermal analysis, electron microscopy, optical microscopy, and Raman spectroscopy. The in vitro aerosol dispersion performance of spray dried TMP was tested using three different FDA-approved unit-dose capsule-based human dry powder inhaler devices. In vitro human cellular studies were conducted on pulmonary cells from different regions of the human lung to examine the biocompatibility and non-cytotoxicity of TMP. Furthermore, the efficacy of inhaled TMP as both liquid and dry powder inhalation aerosols was tested in vivo using the monocrotaline (MCT)-induced PH rat model.

## 1. Introduction

Pulmonary hypertension (PH) is a serious life-threatening disease of the pulmonary and cardiovascular system [1]. It is one of the most devastating chronic diseases of the pulmonary circulation, which can be encountered by healthy individuals living in higher altitude, divers, mountain climber, athletes, and during exercise and rehabilitation [2]. It can also be found in patients in the setting of collagen vascular disease (e.g., localized cutaneous systemic sclerosis), portal hypertension, congenital left-to-right intracardiac shunts, infections with the human immunodeficiency virus (HIV), and persistent pulmonary hypertension of the newborn [3]. PAH can be defined clinically as a mean pulmonary arterial pressure of ≥25 mmHg at rest or ≥30 mmHg during exercise [4,5]. It can be attributed to combined effects of sustained vasoconstriction, concentric vascular remodeling, in situ thrombosis, and arterial wall stiffening, resulting in elevated pulmonary vascular resistance [5]. As a consequence, elevated pulmonary vascular resistance increases the right heart afterload and in the fullness of time results in right ventricular hypertrophy and eventually right heart failure and death [5].

The current therapies fall into several classes, including vasodilators, anticoagulants, antiplatelet agents, anti-inflammatory, and vascular-remodeling therapies [1,3]. Among these are vasodilative and antiproliferative drugs such as nitric oxide (NO), NO-donors, adenosine, calcium channel blockers (e.g., nifedipine, diltiazem), endothelin receptor antagonist (e.g., ambrisentan, bosentan, macitentan), phosphodiesterase inhibitors (sildenafil, tadalafil, vardenafil), prostacyclin and prostacyclin analogs (e.g., beraprost, epoprostenol, iloprost, and 2reprostinil), and guanylate cyclase stimulators (e.g., riociguat) [6]. Most of these drugs are administered to the patient either orally or intravenously leading to systemic side effects such systemic hypotension. The exceptions are two drugs, iloprost and 2reprostinil, which are inhaled therapies FDA-approved for human use in marketed pharmaceutical products as liquid inhalation aerosols delivered by nebulization [7]. There is a great unmet need to have more inhalation aerosol therapeutic drug delivery systems for targeted pulmonary delivery for the treatment of PH.

Tetramethylpyrazine (TMP) (also known as Ligustrazine) is an active alkaloid contained in the rhizome of *Ligusticum chuanxiong* Hort, an herb that has been used for many years in China and Japan as an intravenous (i.v.) infusion solution for the treatment of occlusive cerebral arteriolar disease [8] and PAH [8,9]. It is a vasodilator [10] and its mechanism of action include calcium antagonism, cAMP production, and endothelium-dependent NO-mediated relaxation [9]. Furthermore, it has been reported that TMP could significantly decrease pulmonary hypertension caused by acute and chronic hypoxia in rats and ferrets in ex vivo isolated perfused lung model [11,12]. TMP has been shown to possess neuroprotective [13,14] and anti-platelet functions [15]. The anti-inflammatory effect of TMP resulted in alleviating asthma in rodent models [15,16]. TMP has been shown to have protective lung effects against injury in acute lung injury (ALI) in a rat model of ALI [17]. The inhibitory effect of TMP on the RhoA/ROCK pathway [17,18] is a uniquely distinct pharmacological mechanistic advantage in using TMP to treat PH. The protective effect of TMP on endothelial dysfunction has been reported based on studying oxidative damage and mitochondrial dysfunction [19]. This antioxidant was shown to penetrate the skin when formulated for transdermal drug delivery for Alzheimer’s treatment [20]. In addition, it has been shown to act as a Nrf2 activator (i.e., activates the Nrf/ARE pathway) in oxidative stress injury in vivo in rats [21]. Ligustrazine’s antioxidant effects on lung fibrosis and ROS-related autophagy in an in vivo lung fibrosis model has been reported [22]. The antioxidant effects of ligustrazine in a pulmonary damage burn model has been studied [23].

Off-target systemic side effects are therapeutic-limiting due to systemic administration of the drug as an intravenous (i.v.) infusion acting on the systemic vascular circulatory system. Hence, a targeted approach to PH therapy would be to deliver the drug locally to the airways targeting the lung directly. Whereas i.v. drug administration is invasive, not patient-friendly, and causes a systemic high concentration in a short period of time leading to adverse side effects, localized pulmonary delivery by inhalation aerosols is non-invasive, patient-friendly, and would inherently require a reduced amount of drug to bring about the same therapeutic response [24,25]. Currently, Ventavis^®^ (nebulized iloprost inhalation solution) and Tyvaso^®^ (nebulized treprostinil inhalation solution) are the only FDA-approved inhalation products indicated for the treatment of PH. Ventavis^®^ is delivered as a liquid aerosol through the I-neb nebulizer system and is used to treat both adult and pediatric populations [26]. Relatively long administration times for reaching the needed dose and the high frequency of dosing administration can lead to patient non-compliance with nebulized liquid inhalation products.

Hence, targeted pulmonary drug delivery by inhalation aerosol delivery is not only non-invasive but offers many significant advantages such as a large surface area for high drug absorption [27,28,29], a rapid onset of therapeutic action [24,30], low enzymatic activity [28,29,31], extensive blood supply [32], avoidance of first-pass metabolism [27,28,32], reduced dosing frequency [28], and reduced side effects [27,29,32]. There are added advantages unique to dry powder inhalers which are reduced administration time, reduced frequency of dosing administration, better physicochemical drug stability, more devices available that are versatile, small, lightweight, and portable without needing a power supply. Marketed DPI pharmaceutical products are approved by the FDA for use in children as young as 4 years old. In addition, the top-performing inhalation products on the market are dry powder inhalers due to clinically superior efficacy and high patient acceptance.

In this study, we report on the comprehensive characterization of the solid-state physicochemical properties of raw TMP and advanced spray dried TMP particles. In addition, this study reports on the in vitro aerosol dispersion properties of TMP dry powder aerosols using three different human dry powder inhaler devices varying in shear stress properties and in vitro cellular studies using human lung cells from different regions of the lung. Moreover, this study reports for the first time on the in vivo safety of inhaled TMP in healthy rats and in vivo efficacy of both liquid and dry powder inhalation aerosols in reducing the pulmonary arterial pressure in the validated monocrotaline (MCT) rat model of PH.

## 2. Experimental: Materials and Methods

### 2.1. Materials

**Figure 1:**
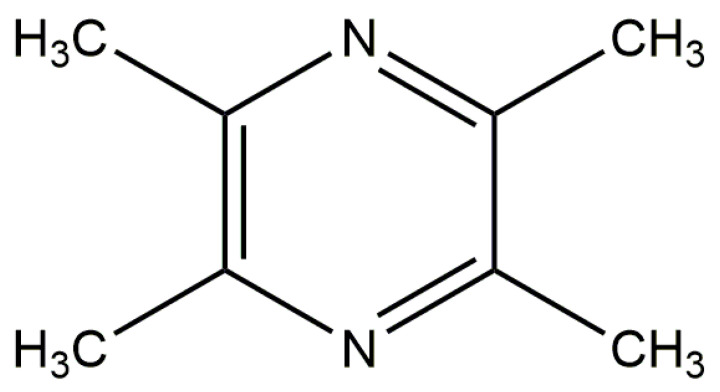
Chemical structure of tetramethylpyrazine (TMP) (ChemDraw^®^ Ultra Ver. 10.0; CambridgeSoft, Cambridge, MA, USA).

TMP (≥98% purity) (C_8_H_12_N_2_; molecular weight (MW): 136.19), shown in Figure 1 (ChemDraw™ Ultra Ver. 10.0; CambridgeSoft, Cambridge, MA, USA), was obtained from Sigma-Aldrich (St. Louis, MO, USA). Methanol (HPLC grade, ACS-certified grade, purity 99.9%) was obtained from Fisher Scientific (Fair Lawn, NJ, USA). Hydranal^®^-Coulomat AD was from Sigma-Aldrich (St. Louis, MO, USA). Resazurin sodium salt and DMSO LC-MS grade were from Thermo Scientific (Waltham, MA, USA). The nitrogen gas used was ultra-high purity (UHP) (Cryogenics and gas facility, The University of Arizona, Tucson, AZ, USA).

### 2.2. Methods

#### 2.2.1. Advanced Closed-Mode Spray Drying from Organic Solution

Spray drying (SD) was carried out using a B-290 Buchi mini spray dryer coupled with a B-295 inert loop and high-performance cyclone (Buchi Labortechnik AG, Flawil, Switzerland) in a closed-mode using compressed UHP dry nitrogen as the atomizing gas. The feed solution was prepared by dissolving 1% *w*/*v* drug TMP in methanol. A stainless steel nozzle with a diameter of 0.7 mm was used to atomize the drug solution. As listed in Table 1, the following conditions were used: atomization gas flow rate 670 L/h (55 mm), aspirator rate of 38 m^3^/h (100%), inlet temperature of 100 °C and feed rate of 27 mL/min. Spray-dried powders were stored in desiccators at −20 °C until further analysis.

#### 2.2.2. Scanning Electron Microscopy (SEM)

Using similar conditions reported in previous studies [24,33], the drug particles as supplied (raw) and spray dried particles were imaged using SEM FEI Inspect S (Brno, Czech Republic) scanning electron microscope. Briefly, the samples were sputter coated with gold for 1.5 min and 15 mAmps current using Anatech Hummer 6.2 (Union City, CA, USA) gold coater and imaged using 30 kV accelerating voltage electron beam.

#### 2.2.3. Laser Diffraction Particle Sizing and Size Distribution

Particle size and size distributions of the particles were determined by laser diffraction with the SALD-7101 (Shimadzu Scientific Instruments, Columbia, MD, USA) using conditions previously reported [34,35,36,37] for measurement of the mean size and size distribution of SD particles in aqueous suspension. Samples were dispersed in ultrapure water and ultrasonicated for 10 s in a water bath ultrasonicator (Branson 7500, Branson Ultrasonics, Danbury, CT, USA) before measuring particle size. The sample particle dispersion was immediately transferred to particle size measuring cell and kept stirring during measurement in nanoparticle size analyzer. The low refractive index of 1.50–0.00 i was used. Volume-based measurements obtained D_V10_, D_V50_, and D_V90_ were used as particle size characterization parameters. The Span value was calculated using the Equation (1) defined as:

Span = [(D_V90_ − D_V10_)/D_V50_] (1)

#### 2.2.4. X-ray Powder Diffraction (XRPD)

Using similar conditions reported by previous studies [24,33], X-ray powder diffraction (XRPD) patterns of samples were collected with Cu Kα radiation (45 kV, 40 mA, and λ = 1.5406 Å) between 10.0° and 80.0° (2θ) with a scan rate of 2.00° per minute at ambient temperature using PanAnalytical X’pert diffractometer (PANalytical Inc., Westborough, MA, USA).

#### 2.2.5. Differential Scanning Calorimetry (DSC)

Thermal analysis and phase transitions measurements were performed using differential scanning calorimeter (DSC) TA Q1000 differential scanning calorimeter (DSC) (TA Instruments, New Castle, DE, USA) with RSC90 automated cooling system similar to previous conditions [24,33]. Samples weighing 1–3 mg of the test powder were used with a hermetic T-Zero^®^ DSC pans for melting (T_m_) and enthalpy (ΔH) measurements. The pans were heated between 0 °C to 150 °C at a heating scanning rate of 5.00 °C/min. All measurements were made in triplicate.

#### 2.2.6. Hot-Stage Microscopy (HSM) under Cross-Polarizers

Using similar conditions reported previously [24,33], hot-stage microscopy (HSM) was performed using Leica DMLP cross-polarized microscope (Wetzlar, Germany) equipped with a Mettler FP 80 central processor heating unit and Mettler FP82 hot stage (Columbus, OH, USA). Powder samples were mounted on a cover glass slide and heated from 25.0 °C to 100.0 °C at a heating rate of 5.00 °C/min. Images were captured periodically.

#### 2.2.7. Karl Fischer Titration (KFT)

The residual water content of spray dried powder was analytically quantified by Karl Fisher (KF) coulometric titration using a TitroLine 7500 trace titrator (SI Analytics, Yellow Springs, OH, USA) similar to previous studies [24,33]. Approximately 1–10 mg of powder was added directly into the reaction cell that contained Hydranal^®^ coulomat AD reagent.

#### 2.2.8. Raman Spectroscopy

Spectroscopy of the raw and spray-dried powders was carried by-non-invasive and non-destructive Raman spectroscopy for molecular fingerprinting with a Renishaw InVia Reflex (Gloucestershire, UK) at the surface using a 20× magnification objective on a Leica DM2700 optical microscope (Wetzlar, Germany) and equipped with a Renishaw inVia Raman system (Gloucestershire, UK) using similar conditions previously reported [24,33,38,39]. Raman spectra were obtained at 514 nm laser excitation using a 20× magnification objective lens.

#### 2.2.9. In Vitro Aerosol Dispersion Performance

In vitro aerosol dispersion study was conducted in accordance with US Pharmacopeia (USP) Chapter <601> specification [40] on aerosols and using conditions similar to those previously reported [24,33]. The Next-Generation Impactor^®^ (NGI^®^) (Copley Scientific, Nottingham, UK) with a stainless steel induction port (USP throat) attachment was used at a flow rate of 60 L/min with an actuation time of 10 s through the inhaler device. The aerosol dispersion performance was tested using three different FDA-approved human unit-dose capsule-based inhaler devices varying in shear stress properties. These three human DPI devices are namely: a low-shear stress device, the Aerolizer^®^ (Merck, Whitehouse Station, NJ, USA); a medium-shear stress device, the Neohaler^®^ (Sunovion Pharmaceuticals Inc, Marlborough, MA, USA); and a high-shear stress device, the HandiHaler^®^ (Boehringer Ingelheim, Ingelheim, Germany). Quali-V clear HPMC size 3 inhalation-grade capsules (Qualicaps, RTP, NC, USA) were filled with about 10 mg of powder. Critical quality attributes of aerosols such as fine particle dose (FPD), fine particle fraction with respect to nominal dose (FPFND), fine particle fraction with respect to emitted dose (FPFED), and emitted dose (ED) were calculated using the following Equations (2)–(5):

(2) FPD=Mass of particles deposited on stages 2 to 7

(3) $$\mathrm{FPFND}=\;\frac{\mathrm{FPD}}{\mathrm{Initial}\;\mathrm{particle}\;\mathrm{mass}\;\mathrm{loaded}\;\mathrm{in}\;\mathrm{capsules}}\;\times \;100\%$$

(4) $$\mathrm{FPFED}=\;\frac{\mathrm{FPD}}{\mathrm{Total}\;\mathrm{particle}\;\mathrm{mass}\;\mathrm{on}\;\mathrm{all}\;\mathrm{stages}}\;\times \;100\%$$

(5) $$\mathrm{ED}=\frac{\mathrm{Initial}\;\mathrm{mass}\;\mathrm{in}\;\mathrm{capsules}\;-\;\mathrm{Final}\;\mathrm{mass}\;\mathrm{remaining}\;\mathrm{in}\;\mathrm{capsules}}{\mathrm{Initial}\;\mathrm{mass}\;\mathrm{in}\;\mathrm{capsules}\;}\;\times \;100\%$$

#### 2.2.10. In Vitro Human Cell Viability

Human pulmonary cell lines for cellular studies were purchased from the American type culture collection ATCC^®^ A549 (ATCC^®^ CCL-185™), H358 (ATCC^®^ CRL-5807™) and Calu-3 (ATCC^®^ HTB-55™). A549 and H358 were grown in Dulbecco’s modified eagle’s medium (DMEM) Advanced 1X supplemented with fetal bovine serum (FBS), Pen-Strep, Fungizone^®^, and L-glutamine obtained from Gibco^®^ by Life Technologies (Thermo Fisher Scientific Inc., Hanover Park, IL, USA). Calu-3 cell line was grown in Eagle’s minimum essential medium (EMEM) obtained from ATCC supplemented with FBS, Pen-Strep and Fungizone^®^ obtained from Gibco^®^ by Life Technologies (Thermo Fisher Scientific Inc., USA).

Cell-based assays are often used to determine if test molecules have effects on cell proliferation or cell death. The A549 pulmonary cell line is a human alveolar epithelial lung adenocarcinoma cell line for the deep lung small airways (respiratory region) and often used as an in vitro human cellular model of the alveolar type II pneumocyte cell in in vitro pulmonary drug delivery and metabolism studies. The H348 pulmonary cell line is a human bronchioalveolar epithelial cell line from the deep lung small airways (respiratory region) and expresses lung surfactant-associated protein A (SP-A) [25]. Both cell lines were grown in a growth medium including Dulbecco’s modified eagle’s medium (DMEM) advanced 1×, 10% (*v*/*v*) fetal bovine serum (FBS), Pen-Strep (100 U/mL penicillin, 100 μg/mL), Fungizone (0.5 μg/mL amphotericin B, 0.41 μg/mL sodium deoxycholate), and 2 mM L-Glutamine in a humidified incubator at 37 °C and 5% CO_2_.

As previously reported [41], both cell lines were seeded in 96-well plates at 5000 cells/well and 100 μL/well and allowed to attach for 48 h. Then, the cells were exposed to 100 μL of TMP dissolved in media at different concentrations and incubated for 72 h after exposure. Continuously, 20 μL of 10 μM resazurin sodium salt dissolved in 1% DMSO in media was added to each well and incubated for 4 h. At this point, the fluorescence intensity was detected at 544 nm (excitation) and 590 nm (emission) using a Synergy H1 Multi-Mode Reader (BioTek Instruments Inc., Winooski, VT, USA). The relative viability of each sample was calculated as follows:

(6) $$\mathrm{Relative}\;\mathrm{viability}\;\left(\%\right)=\;\frac{\mathrm{Sample}\;\mathrm{fluorescence}\;\mathrm{intensity}\;}{\mathrm{Control}\;\mathrm{fluorescence}\;\mathrm{intensity}\;}\times \;100\%$$

#### 2.2.11. In Vitro Transepithelial Electrical Resistance (TEER)

Calu-3 lung epithelial cells, a human lung adenocarcinoma cell line derived from the bronchial submucosal large airway region, were grown in a growth medium including Eagle’s minimum essential medium (EMEM) (obtained from ATCC), 10% (*v*/*v*) fetal bovine serum (FBS), Pen-Strep (100 U/mL penicillin, 100 μg/mL), Fungizone (0.5 μg/mL amphotericin B, 0.41 μg/mL sodium deoxycholate) in a humidified incubator at 37 °C and 5% CO_2_, as previously reported [24]. The cells were seeded at 500,000 cells/mL in Costar Transwells^®^ (0.4 μm polyester membrane, 12 mm for a 12-well plate, Corning, NY, USA) with 0.5 mL of media on the apical side and 1.5 mL of media on the basolateral side. Media was changed every other day from the basolateral and apical side. After 10 days of growth, when the cells reached a TEER value of about 1000 Ω/cm^2^ which is an indicator of a confluent monolayer at liquid covered culture (LCC) the media was removed from both sides and 800 μL of media was added to the basolateral side of the transwells to facilitate air-interface culture (AIC) conditions. The TEER responses of the cells were measured with an Endohom 12 mm Culture Cup (World Precision Instruments, Sarasota, FL, USA). For TEER measurement, 0.5 mL of media was added to the apical side of the Transwell 5 min before measurement and then immediately removed to return the cells to AIC conditions. After the TEER values reached 500 Ω/cm^2^ (indicating a confluent monolayer at AIC conditions), the cells were exposed to 100 μM of SD TMP and representative co-SD formulations dissolved in non-supplemented EMEM media. The liquid aerosol formulations were delivered to the Calu-3 cells at AIC using the Penn Century MicroSprayer^®^ Aerosolizer-Model IA-1B. TEER values were then recorded for up to 7 days after aerosol treatment, as previously reported [24,42].

#### 2.2.12. In Vivo Efficacy in PH-Induced Rats

In vivo studies were conducted in the monocrotaline (MCT) rat model of PH, a validated in vivo PH disease animal model, for testing inhaled TMP efficacy in decreasing PH. TMP was administered as an inhaled liquid aerosol in one study and as an inhaled spray-dried powder aerosol in a separate study. All rats were purchased from Charles River Laboratories International Inc. (Wilmington, MA, USA) and were weighed weekly to monitor their health. PH was induced by injecting 60 mg/kg of MCT dissolved in phosphate buffered saline (PBS) intraperitoneally (IP injection).

Inhaled liquid aerosol study description: A total of 25 male Sprague Dawley rats, each weighing in the range of 260–294 g, were used in this study. The animals were divided into 5 groups (*n* = 5) into the following categories: Group 1 Naïve (control); Group 2 Naïve + TMP; Group 3 Naïve + vehicle (drug-free solvent); Group 4 MCT (PH-induced); and Group 5 MCT + treated with TMP. Two weeks after MCT administration, the rats began inhaled aerosol treatment of an aerosolized liquid formulation of TMP using the Penn-Century MicroSprayer^®^ Aerosolizer-Model IA-1B (Penn-Century, Inc, Wyndmoor, PA, USA) for 14 days. Each animal received 500 microliters of liquid TMP formulation. For the first few days, the animals received a dose of 100 mg/kg of TMP dissolved in 100% ethanol vehicle, but there were some unforeseen death in the study. Hence from day 4, the remaining animals received a dose of 10 mg/kg of TMP dissolved in a vehicle comprised of 10% ethanol + 90% PBS. The vehicle group animals received the respective solvent systems used to dissolved TMP. Right ventricle systolic pressure (RVSP) hemodynamics were performed at the end of 14 days drug treatment by inserting a customized pressure transducer catheter (SPR-513, Millar Instruments, Houston, TX, USA), into the right ventricle (RV) via the right jugular vein and right atrium. The transducer was connected to a Millar Transducer Control Unit TC-510 and PL3504 PowerLab 4/35 data acquisition system (AD Instruments, Inc., Colorado Springs, CO, USA). At the end of pressure recording, the animals were euthanized.

Inhaled spray-dried powder aerosol study description: A total of 30 male Sprague Dawley rats, each weighing in the range of 280–350 g, were used in this study. The animals were divided into 5 groups (*n* = 5) into the following categories: Group 1 Naïve (control); Group 2 Naïve + TMP; Group 3 MCT (PH-induced); Group 4 MCT+ treated with vehicle (air); and Group 5 MCT + treated with TMP. Two weeks after MCT IP injection, the rats started to receive treatment via aerosolization of a dry powder formulation of SD TMP (10 mg/kg dose) using the Penn-Century Dry Powder Insufflator™-Model DP-4R (Penn Century Inc., Wyndmoor, PA, USA), the dry powder inhaler delivery device customized for the respiratory tract of the rat, for 14 days. RVSP hemodynamics measurements were made similar to the liquid aerosol study. However, the measurements were performed at 2 weeks and 4 weeks after MCT administration to establish that the disease was induced. At the end of pressure recording, the animals were euthanized.

All animal studies were performed in accordance with Institutional Animal Care & Use Committee (IACUC) guidelines for the care and use of laboratory animals under the protocol approved by University of Arizona Institutional Animal Care & Use Committee.

#### 2.2.13. Statistical Analysis

All experiments were performed in at least triplicate (*n* = 3) unless otherwise mentioned. All data were statistically analyzed using Sigma Plot 13.0 (Systat Software Inc., San Jose, CA, USA). Unpaired student t-test and one-way analysis of variance (ANOVA) were performed to compare the statistical significance of the test groups. *p* value ≤ 0.050 was considered to be statistically significant.

## 3. Results

### 3.1. Scanning Electron Microscopy (SEM)

**Figure 2:**
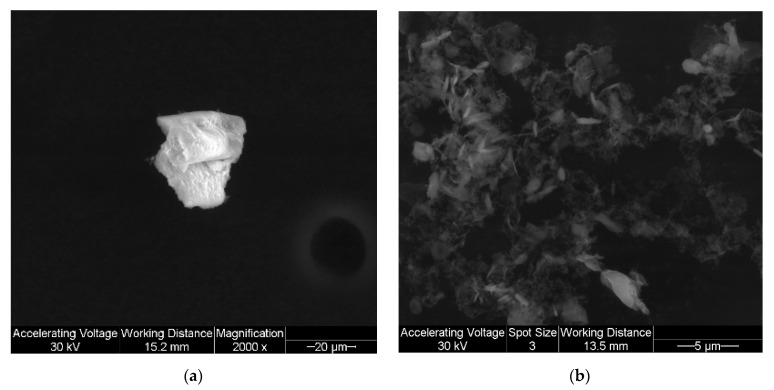
Scanning electron micrographs of: (**a**). raw TMP (magnification 2000×) and (**b**). SD TMP (magnification 10,000×).

The particle shape and surface morphology were visualized by SEM as shown in Figure 2 for raw TMP and SD TMP. SD TMP exhibited oblong and relatively smooth morphology compared to raw TMP.

### 3.2. Laser Diffraction Particle Sizing

Laser diffraction sizing experiments were carried out on spray dried particles. The particles size distribution from the laser diffraction was in the range of 4–14 μm. The average values of Dv10, Dv50 and Dv90 were 4.186 ± 0.701, 6.156 ± 1.47 and 14.552 ± 4.928 μm, respectively. The Span value was calculated to be 1.684 ± 0.645.

### 3.3. X-ray Powder Diffraction (XRPD)

**Figure 3:**
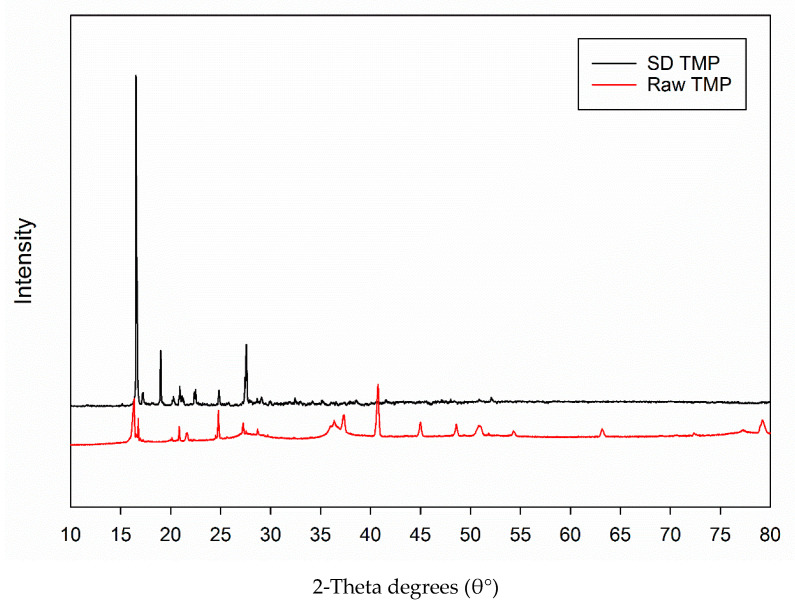
X-ray powder diffractograms of raw TMP and SD TMP.

The XRPD pattern of raw TMP showed sharp and intensive peaks at 2θ-degree values of 16.36°, 24.78°, 36.35°, 37.37°, 40.75°, 45.03°, 48.58° and 50.89° characteristic of long-range molecular order (i.e., crystallinity) as shown in Figure 3. SD TMP had intensive peaks at 16.56°, 19.00°, 24.83°, and 27.56° indicative of the retention of crystallinity following spray drying. It is notable that the SD TMP particles, unlike raw TMP, did not exhibit any peak in the 2θ region of 30–60°. This is suggestive of a possible polymorphic conversion from the raw TMP crystalline form following spray drying under these conditions.

### 3.4. Differential Scanning Calorimetry (DSC)

**Figure 4:**
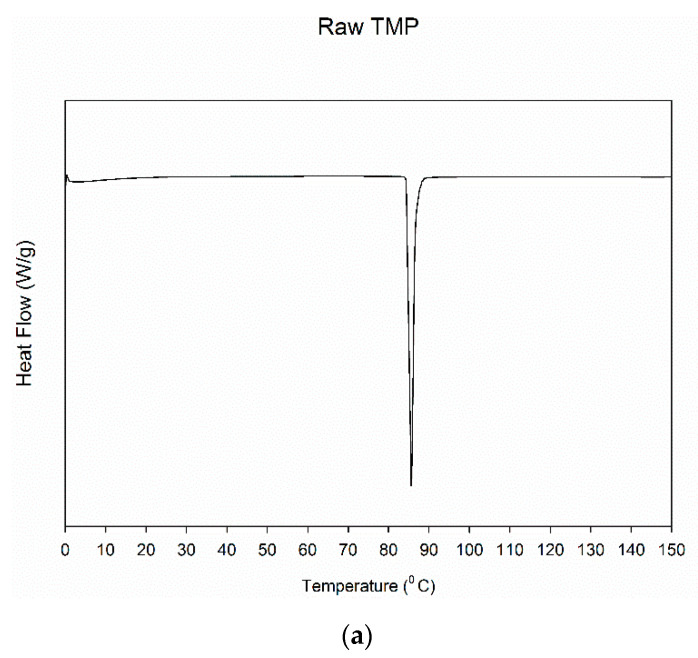

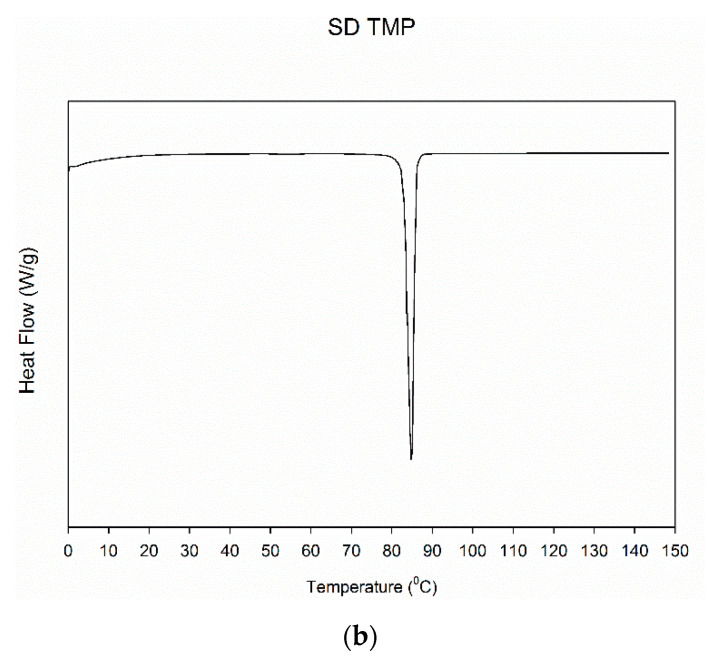
DSC thermograms of: (**a**) Raw TMP and (**b**) SD TMP (Exothermic peak is upward; Endothermic peak is downward).

DSC of the raw TMP and spray dried TMP showed in Figure 4 has a single transition at around 85 °C. The single endotherm seen in the thermogram suggests that TMP is crystalline with a melting point around 85 °C.

### 3.5. Hot-Stage Microscopy (HSM) under Cross-Polarizer Lens

**Figure 5:**
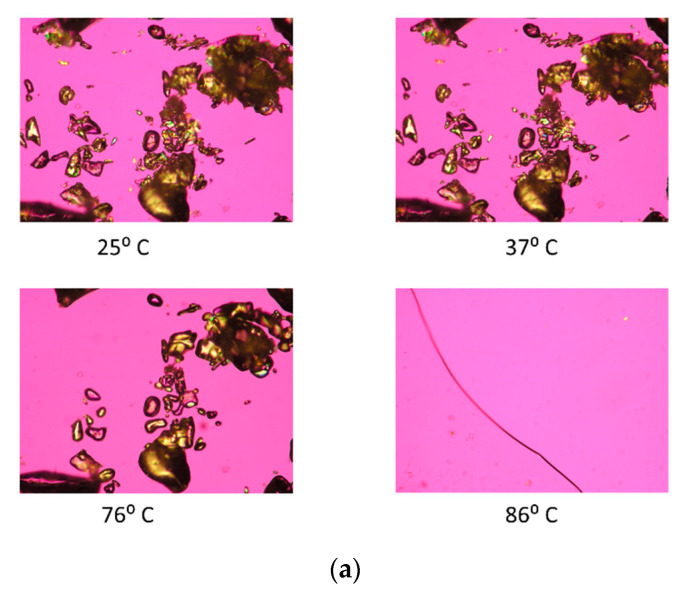

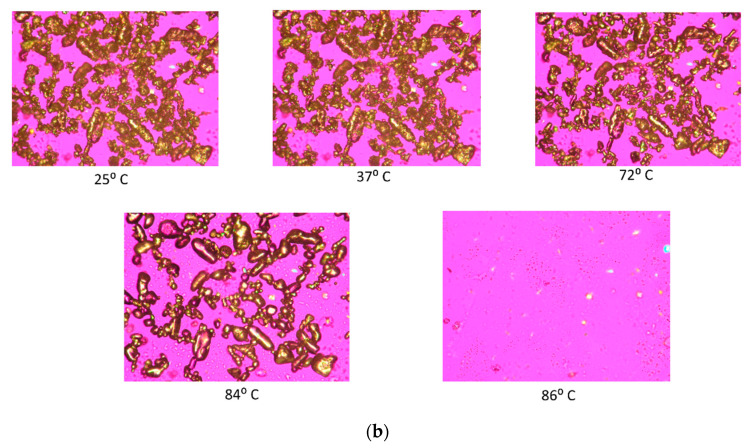
Hot-stage micrographs of (**a**) Raw TMP and (**b**) Spray Dried (SD) TMP.

Raw TMP and SD TMP particles were visualized under cross-polarized light, as seen in Figure 5, and both exhibited birefringence, which is a characteristic feature of crystals. Upon heating the particles at a constant heating rate, raw TMP particles started melting at approximately 62 °C and completely melted at approximately 86 °C. While the SD TMP particles started melting around 60 °C and completely melted around 86 °C. This is consistent with DSC thermal analysis.

### 3.6. Karl Fischer Titration (KFT)

The residual water content in the powder using Karl Fischer titration was 0.633 ± 0.251% *w*/*w* for raw TMP. However, the SD TMP had a lower water content of 0.368 ± 0.103% *w*/*w* which can be attributed to the spray drying process. Table 2 lists the KFT values for raw and SD TMP powders.

### 3.7. Raman Spectroscopy

**Figure 6:**
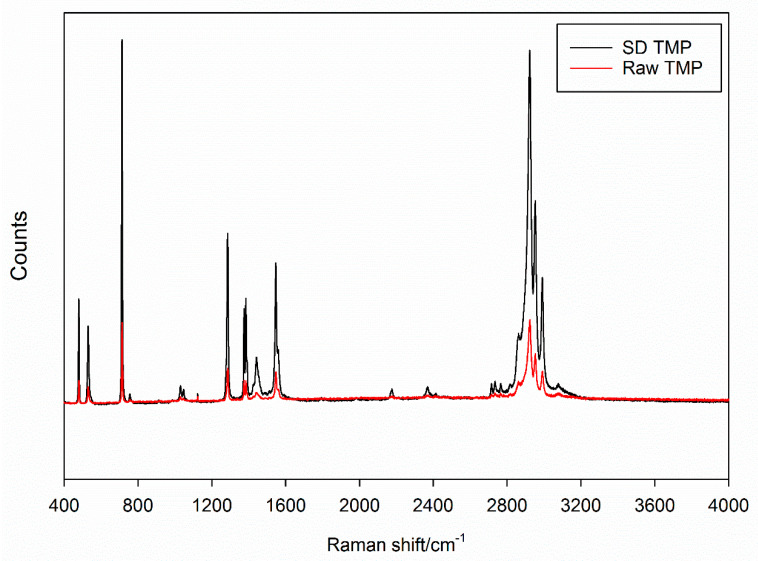
Raman spectra of raw TMP and spray dried (SD) TMP.

From Figure 6, the raw TMP and SD TMP have similar molecular fingerprinting spectra suggesting that the TMP molecule remained the same before and after spray drying. Prominent peaks were seen at 714 cm^−1^, 1286 cm^−1^, 1547 cm^−1^ and between 2900–3000 cm^−1^ wavenumbers. The sharp peaks seen in this spectra concurs with the inference of TMP crystallinity before and after spray drying.

### 3.8. In Vitro Aerosol Dispersion Performance

**Figure 7:**
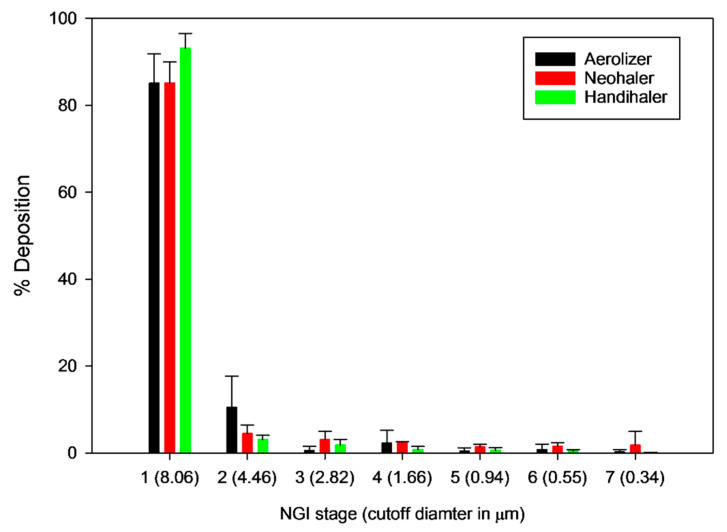
In vitro aerosol deposition of spray dried TMP particles using human dry powder inhaler (DPI) devices Aerolizer^®^, Neohaler^®^ and Handihaler^®^ using NGI^®^ at 60 L/min flow rate. (*n* = 3, mean ± SD).

The aerosol dispersion performance of SD TMP using the three unit-dose capsule-based inhaler devices with varying shear stress properties is listed in Table 3. The NGI stage deposition using different human DPI devices is exhibited in Figure 7. The ED from the high-resistance HandiHaler^®^, (Boerhinger Ingelheim, Ingelheim, Germany) device was 100%, while the other two devices emitted 88% of the loaded powder from the capsules. The FPFND values were 1.41%, 4.36% and 3.33% for the Aerolizer^®^ (Novartis AG, Basel, Switzerland), Neohaler^®^ (Novartis Pharma Stein AG, Stein, Switzerland), and HandiHaler^®^ (Boerhinger Ingelheim, Ingelheim, Germany), respectively.

### 3.9. In Vitro Human Cell Viability

**Figure 8:**
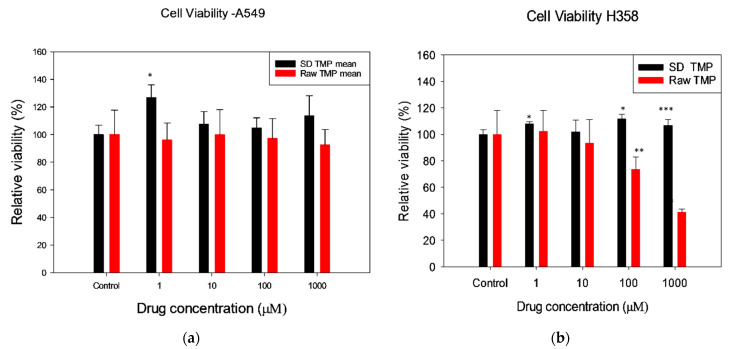
In Vitro Vell Viability of: (**a**). A549 and (**b**). H358 human pulmonary cell lines. * *p* value < 0.001, ** *p* value 0.010, *** *p* value 0.015. (*n* = 6, Mean ± SD).

In vitro cell viability assay was performed on pulmonary cell lines A549 and H358. Molar concentrations of 1 μM, 10 μM, 100 μM, and 1000 μM of raw TMP and SD TMP were tested after dissolving the drug in media. The relative cell viability of A549 cell line was 100% for both raw TMP and SD TMP at all concentrations. The relative viability of H358 cell line was 100% for SD TMP, however, at higher concentrations, the viability was decreased. The cell viability using different drug concentrations is shown in Figure 8.

### 3.10. In Vitro Transepithelial Electrical Resistance (TEER)

**Figure 9:**
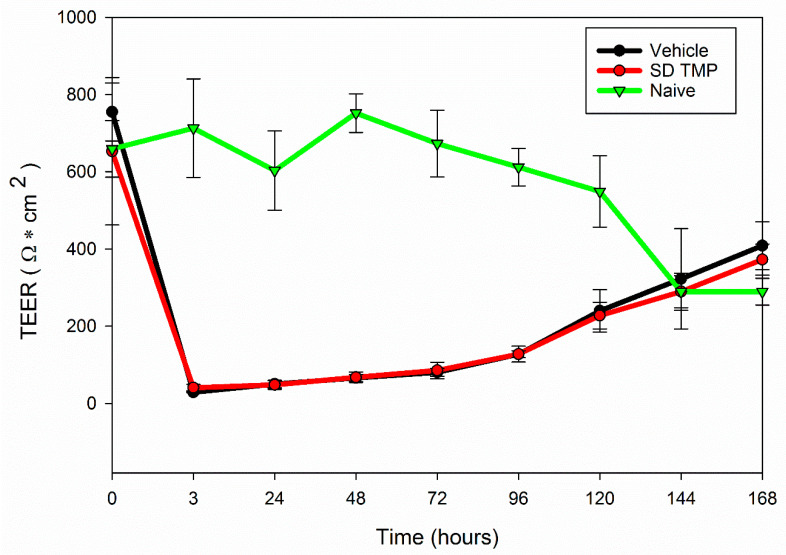
In Vitro transepithelial electrical resistance (TEER) measurements using human Calu-3 bronchial epithelial cell line exposed to representative liquid aerosol formulations in air-interface culture (AIC) conditions using Microsprayer^®^ Aerosolizer. (*n* = 3, mean ± SD).

It can be seen from Figure 9 that three hours after treatment of the cells with 100 μM of SD TMP as a liquid aerosol using the Microsprayer^®^ Aerosolizer (Penn-Century, Wyndmoor, PA, USA), the electrical resistance decreased, however on continuous culturing for seven days following the treatment, the resistance was regained. Calu-3 cells treated with the vehicle (10% ethanol + 90% EMEM non-supplemented media) had a similar trend of decreased resistance three hours after liquid aerosol treatment, followed by gradual increase in the resistance over a period of time.

### 3.11. In Vivo Efficacy Study

**Figure 10:**
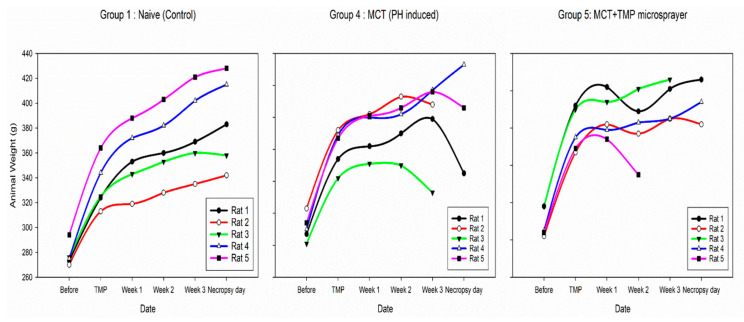
Body weights of rats measured weekly for Groups 1, 4 and 5 from Microsprayer^®^ Aerosolizer (liquid aerosol) study.

**Figure 11:**
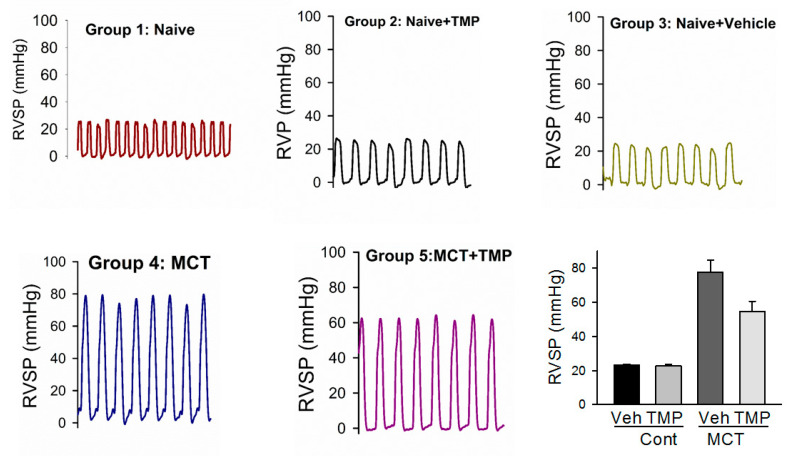
Representative in vivo right ventricle systolic pressure (RVSP) measurements of different rat groups from Microsprayer^®^ Aerosolizer (liquid aerosol) study.

The rat’s body weight plots in Figure 10 shows a loss of weight with time for the Groups 4 and 5 that received MCT. In contrast, the Group 1 (Naïve) rats showed a steady increase in body weight with time (Figure 10). As it is presented in Figure 11 and Table 4, the RVSP measurements in Group 1 (Naïve), Group 2 (Naïve+ TMP) and Group 3 (Naïve+ vehicle) are ranging between 20–25 mmHg (normal RV pressure in rats). Four weeks after MCT administration, the RVSP increased more to 80–100 mmHg in the untreated Group 4 suggesting that the animals had severe PH. As can be seen from Figure 11, after four weeks of treatment with TMP as liquid aerosol (Group 5) the RVSP significantly decreased by 20–30 units of mmHg relative to Group 5 (4 weeks).

**Figure 12:**
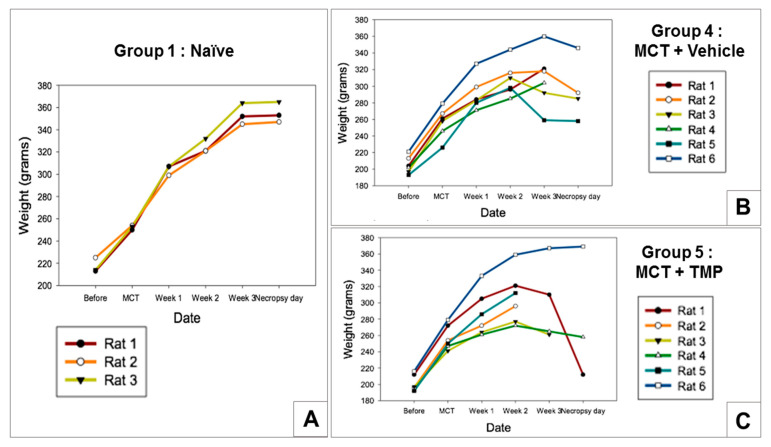
Body weights of rats measured weekly for: (**A**) Group 1; (**B**) Group 4; and (**C**) Group 5 from insufflator (dry powder aerosol) study.

**Figure 13:**
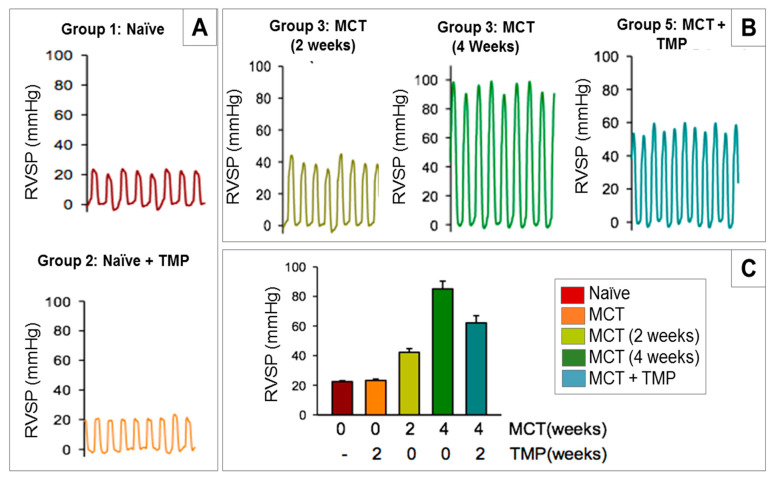
Representative in vivo right ventricle systolic pressure (RVSP) measurements of: (**A**). Groups 1 & 2; (**B**) Groups 3 & 5; and (**C**) pressure measurement comparison between different rat groups from insufflator (dry powder aerosol) study.

The rat body weight plots in Figure 12 clearly show a significant loss of weight with time for the Groups 4 and 5 that received MCT. In contrast, Group 1 (Naive) rats showed a steady increase in body weight with time (Figure 12). As shown in Figure 13 and Table 5, the RVSP hemodynamic measurements in Groups 1 (Naïve) and 2 (Naïve+ TMP) are ranging between 20–25 mmHg (normal RV pressure in rats). Two weeks after MCT administration, the RVSP considerably increased to a range of 40–50 mmHg, as can be seen in Figure 13B. Four weeks after MCT administration, the RVSP increased even more to a range of 80–100 mmHg in the untreated Group 3. It is evident from the Figure 13B hat after two weeks of aerosol treatment with SD TMP (Group 5) the RVSP significantly decreased by ~20–30 mmHg relative to Group 3 (4 weeks). It is important to note that the insufflator device used to administer the powder had no effect on the RVSP of Group 4 rats (data not shown).

## 4. Discussion

This is the first report on TMP development and formulation as dry powder aerosols using advanced organic closed-mode spray drying process (Table 1). The spray dried particles were comprehensively characterized to test their suitability for pulmonary drug delivery. From the results obtained in the in vitro human cellular studies on cell viability and TEER seen in Figure 8 and Figure 9, respectively, it is seen that the spray-dried TMP formulation at various concentrations is non-cytotoxic and biocompatible with human pulmonary cells from different regions of the lungs including the respiratory deep lung region. It was observed that possibly a polymorphic conversion occurred following spray drying based on XRPD analysis (Figure 3). This is evident from the lack of peaks in the XRPD diffractogram (Figure 3) between 2θ-degree values of 30–50°. Previously Zhang et al., reported [43] the X-ray diffraction pattern of ligustrazine that is similar to the raw TMP diffraction pattern reported in our present study. However, when TMP was formulated with a PLGA diblock polymeric carrier, the crystallinity changed depending on the extent of miscibility of TMP with PLGA diblock copolymer. Hence, other crystal forms of TMP (i.e., polymorphs) may possibly exist.

In our study, advanced spray drying under the described conditions generally caused a disruption in the crystallinity of TMP often rendering it non-crystalline (i.e., amorphous). Our group has previously reported crystallinity retention by polymorphic interconversion following advanced spray drying [44]. Spray drying is a high-throughput engineering technology that designed and produces the particles tailored for pulmonary inhalation aerosols to be in the desired size range, shape and surface property [27,34,35,39,45,46,47,48]. The particle sizing data using laser diffraction (Table 2) and in vitro NGI aerosol dispersion performance (Figure 7) show that the size range of SD TMP particles readily aerosolize and are in the inhalable size range. The particle morphology is non-spherical and surface morphology is smooth for TMP particles, as seen in the SEM micrographs (Figure 2).

The aerosol dispersion of such particles is expected to be influenced by the shear stress property of the DPI device that is used to generate the aerosol [49,50,51,52,53] and the dry powder interparticulate interactions [30,54]. The DPI device design, specific resistance and shear stress caused by the device have a great impact on the improved or diminished performance of the aerosol formulation [55]. We tested three different FDA-approved human DPI devices to examine the influence of the device type (low-, medium-, or high-shear stress) on in vitro aerosol performance of SD TMP particles. From the NGI impactor data (Figure 7 and Table 3), it can be seen in Figure 7 that the particle deposition in the early stages of NGI is higher than the later stages. One-way ANOVA tests showed statistical significance between the FPFND of Aerolizer^®^ versus HandiHaler^®^ (*p* = 0.006) and Aerolizer^®^ versus Neohaler^®^ (*p* = 0.010). The same test showed no statistical significant difference between the FPFND of Neohaler^®^ when compared with HandiHaler^®^ (*p* = 0.378). Similarly, the ED from the three devices had no statistically significant (*p* = 0.103) differences.

It can be inferred from the mass deposition data that the maximum fine particle dose that can be achieved using this formulation will be 3.48 mg using Neohaler^®^, a medium-shear stress DPI device. The high-shear stress DPI device (HandiHaler^®^) and the medium-shear stress DPI device (Neohaler^®^) achieved similar fine particle doses while the low-shear stress device (Aerolizer^®^) gave a relatively lower FPD. This suggests that, in addition to the turbulence created in the dry powder inhaler, there are other inhaler device factors that can influence the aerosolization and deaggregation of the SD TMP particles.

SD TMP did not significantly affect the cell viability of both A549 and H358 human pulmonary cell lines (Figure 7 and Figure 8) representing the deep lung respiratory region of the lungs. Hence, this suggests that the drug over a wide dose range is non-cytotoxic and biocompatible on these cells. The TEER analysis (Figure 8) showed that the TMP-treated Calu-3 cells regained resistance similarly as the cells treated with the vehicle. There was no statistical difference between the vehicle and the SD TMP. After 7 days, the final resistance reached by all untreated (naïve), vehicle-treated and SD TMP-treated cells were statistically similar. This finding showed that the monolayer recovery happens as early as six days. The loss of electrical resistance and recovery suggests a reversible process, which in turn, ensures the integrity of the cellular barrier and further suggesting non-cytotoxicity and biocompatibility on these cells.

The in vivo data (Table 4 and Table 5, and Figure 10, Figure 11, Figure 12 and Figure 13) showed that the MCT model successfully achieved PH in the rodent after two weeks of administration via intraperitoneal (IP) injection. The RVSP significantly increased after four weeks of MCT administration as it was reported previously [37]. In general, the results from both the insufflator and microsprayer study suggest that TMP is effective in preventing the progression of PH. The study design included a liquid aerosol, in addition to powder, since the dissolution of TMP powder in airway fluids had not been previously reported. Moreover, the influence of the liquid state vs. dry powder state on inhalation treatment efficacy is important.

The body weight plots of the rats showed development of the disease, as can be seen in Figure 10 and Figure 12, where MCT-induced PH rats lost weight with time. In contrast, naïve rats gained weight with time and maintained the weight as it was expected. Regarding the RVSP values (Table 4 and Table 5, Figure 11 and Figure 13), it was noticed that groups 1 and 2 rats with and without drug (TMP) treatment maintained a pressure of ~25 mmHg after four weeks, which is normal in naïve rats. After two weeks of the MCT injection, an increase in the RVSP pressure is observed. After four weeks, the increase in the RVSP reveals severe PH. This was observed in both the liquid and powder aerosol studies indicating that MCT is a suitable model for PH therapeutics study. It is seen in Figure 10 and Figure 12 that the treatment of the rats with inhaled TMP as both powder and liquid aerosols for 2 weeks has a remarkable impact on the significant decrease of RVSP pressure. For both the inhaled liquid aerosol of TMP and the advanced spray-dried DPI of TMP, the RVSP measurements of the drug-treated groups were in the range of 60 mmHg, while the diseased animal model was 80 mmHg. For the first time, this study demonstrates that TMP, when administered as an inhaled aerosol targeting the lungs, can prevent the progression of PH in the MCT-induced PH rat model.

## 5. Conclusions

The physicochemical properties of TMP are reported comprehensively. The in vitro aerosol dispersion performance of spray dried TMP was tested using three different FDA-approved human dry powder inhaler devices. In vitro human cellular studies conducted on pulmonary cells from different regions of the human lung demonstrated biocompatibility and non-cytotoxicity of TMP. The in vivo healthy rat study demonstrated safety of inhaled TMP formulations as liquid aerosols and advanced dry powder aerosols. The in vivo diseased rat MCT model of PH study demonstrated that inhaled TMP aerosols, both as an inhaled liquid and dry powder, are efficacious in the attenuation of PH.

## Figures and Tables

**Table 1:** Advanced spray drying parameters for spray dried (SD) TMP powders using methanol (MeOH).

Feed Concentration (%*w*/*v*)	Pump Rate (% and mL/min)	Inlet T (°C)	Outlet T (°C)
1	90/27	149–152	18–25

**Table 2:** Physical properties of raw TMP and spray dried (SD) TMP. (*n* = 3, Mean ± SD).

Physical Properties	Raw TMP	SD TMP
Particle Sizes	N/A	D_v10_ = 4.186 ± 0.701 μm
		D_v50_ = 6.156 ± 1.147 μm
		D_v90_ = 14.552 ± 4.928 μm
(D_v_)		Span = 1.684 ± 0.645 μm
Phase Transitions	T_m_ = 85.93 ± 0.71 °C	T_m_ = 85.11 ± 0.36 °C
(T_m_ and ΔH)	ΔH = 152.5 ± 5.27 J/g	ΔH = 149.2 ± 7.91 J/g
Residual Water Content	0.633 ± 0.251% *w*/*w*	0.368 ± 0.103% *w*/*w*

**Table 3:** In vitro aerosol dispersion performance parameters for spray dried TMP particles using human dry powder inhaler (DPI) devices Aerolizer^®^, Neohaler^®^ and Handihaler^®^ at 60 L/min. (*n* = 3, Mean ± SD).

Inhaler Device	Emitted Dose (%)	Fine Particle Dose with Respect to Nominal Dose (mg) Fine Particle Fraction with Respect to Nominal Dose (FPFND) (%)	Fine Particle Dose with Respect to Emitted Dose (mg) Fine Particle Fraction with Respect to Emitted Dose (FPFED) (%)
Handihaler^®^	100 ± 2.03	3.33	37.24
		3.83 ± 0.42	8.9 ± 3.87
Neohaler^®^	88.41 ± 6.70	3.48	25.19
		4.36 ± 0.65	14.87 ± 7.30
Aerolizer^®^	88.76 ± 7.27	1.15	8.16
		1.41 ± 0.57	14.86 ± 8.0

**Table 4:** In vivo right ventricle pressure hemodynamic measurements from microsprayer (liquid aerosol) study.

Control + Vehicle Group (mm Hg)	Control + TMP Group (mm Hg)	MCT Group (mm Hg)	MCT + TMP Group (mm Hg)
23.6567	23.8947	94.1697	61.1996
23.7513	22.9558	60.2783	43.1133
20.6622	19.0997	78.0645	60.1284
24.0879	24.6036	78.5773	N/A
23.6379	N/A	N/A	N/A

**Table 5:** In vivo right ventricle pressure hemodynamic measurements from insufflator (powder aerosol) study.

Control (mm Hg)	Control + TMP Group (mm Hg)	MCT (2 Weeks) Group (mm Hg)	MCT (4 Weeks) Group (mm Hg)	MCT + TMP Group (mm Hg)
22.5330	20.8110	39.4080	97.6720	78.2010
21.9020	24.2410	52.0390	95.7920	70.5310
20.9950	23.8990	38.4880	90.0360	58.6830
19.3520	24.7030	41.0640	95.1610	62.4200
22.3220	26.0550	40.3810	79.8910	43.3890
23.6890	19.6940	N/A	78.3780	59.8200

## Data Availability

The data presented in this study are available on request from the Corresponding Author.
